# Using Spatial Technologies to Assess Risk Factors for Diarrheal Disease Under Environmental Variability in Bangladesh: A Machine Learning Study

**DOI:** 10.3390/ijerph22111758

**Published:** 2025-11-20

**Authors:** Ryan van der Heijden, Elizabeth M. B. Doran, Parker King, Kennedy P. Brown, Donna M. Rizzo, Kelsey M. Gleason

**Affiliations:** 1 Department of Civil & Environmental Engineering, University of Vermont, Burlington, VT 05405, USA; ryan.van-der-heijden@uvm.edu (R.v.d.H.); elizabeth.doran@uvm.edu (E.M.B.D.); kennedy.brown@uvm.edu (K.P.B.); donna.rizzo@uvm.edu (D.M.R.); 2 Gund Institute for Environment, University of Vermont, Burlington, VT 05405, USA; plking2@asu.edu; 3 School of Sustainability, Arizona State University, Tempe, AZ 85281, USA; 4 Department of Biomedical and Health Sciences, University of Vermont, Burlington, VT 05405, USA

**Keywords:** spatial analysis, GIS, diarrheal disease, environmental health, climate change, Bangladesh, random forest

## Abstract

Background: Diarrheal disease (DD) remains a major public health challenge and is the leading cause of malnutrition and the second leading cause of death among children under five globally. Although DD can be caused by a wide range of pathogens, its primary drivers are often linked to unimproved sanitation, limited access to clean drinking water, and poor hygiene practices. Low- and middle-income countries, particularly those in South Asia, experience the highest burden. These regions are also increasingly vulnerable to climate change and land use/cover changes, which may further exacerbate DD risk. However, the relative influence of environmental and social drivers at localized scales is not well understood. This gap presents a critical opportunity to identify scalable, data-informed interventions that address environmental determinants of health in the context of a changing climate. Methods: To investigate these dynamics, we analyzed 21,779 records from the Demographic and Health Surveys (DHS) for Bangladesh, integrating them with remotely sensed data on forest cover change, temperature, and rainfall. Using Random Forest machine learning models, we assessed the relative importance of both environmental and socio-demographic variables at household and regional (village) levels. Results: The results show that DD risk varies across scales: household-level outcomes are primarily associated with socio-demographic characteristics, while regional-level outcomes are more strongly influenced by environmental and geographic features, including precipitation, elevation, and proximity to water bodies. Conclusions: These findings underscore the importance of scale-sensitive approaches when assessing environmental health risks and developing climate-adaptive public health strategies.

## 1. Introduction

Control of infectious diseases in the context of environmental change requires novel data collection and analysis methods to reveal the complex connections between disease and the changing environment so that actionable steps may be taken to suppress disease risk. In 2019, diarrheal disease (DD) contributed to over 1.53 million deaths globally and an additional 80.9 million disability-adjusted life years (DALYs) [1]. Yet, the distribution of the global burden of DD is not equal. Almost 90% of the total deaths resulting from diarrheal disease occur in low–middle income countries (LMICs), with the highest proportion of those deaths being children under the age of five [2,3]. In fact, nearly 10% of global deaths attributed to DD are of children under the age of five [4]. The disease also represents the leading cause of malnutrition and because there are multiple pathogens that can cause the disease, it is particularly difficult to study [4]. As the global population continues to grow and as urban centers continue to expand, DD is likely to become more prevalent, placing a greater burden on local hospitals and health centers [5].

The World Health Organization (WHO) further estimates that between 2030 and 2050, climate change, including changes in rainfall patterns, will result in an additional 48,000 deaths per year due to diarrheal disease [6]. The literature on this topic links seasonal trends with diarrheal disease outbreaks, specifically spikes in risk of DD associated with increases in precipitation, although some studies report no significant correlation with high rainfall alone [7,8,9,10]. Additional work has found that increased frequency and intensity of rainfall may have a significant correlation to DD outbreaks. However, dry periods antecedent to heavy rainfall were found to have the highest association with DD risk, which is most likely explained by the “concentration–dilution hypothesis” [9,11]. This mechanism proposes that under dry conditions, there is a higher concentration of pathogens within ground and surface waters resulting from sanitation practices. When rapid rain events occur, surface runoff transports pathogens from highly concentrated sources into the drinking water supply, increasing the risk of exposure to harmful pathogens and DD [12].

Population density, geography and land use, and land cover factors are also important factors in diarrheal disease risk. Studies have found significant differences between DD prevalence in urban versus rural areas where factors such as population density, sanitation practices, and safe drinking water availability can vary widely across the urban-rural continuum [13]. Some studies find land cover change, including forest loss, to be associated with increased DD incidence [12]. Forests and vegetated areas have the ability to impact hydrologic processes that may control pollutant concentration including infiltration which can recharge groundwater and provide baseflow and runoff, which can cause soil erosion and flooding [8,12]. Topography can also influence pathogen exposure levels. Lower-lying regions are particularly susceptible as extreme flooding can result from minor rainfall in these regions. Flooding events, which are more persistent with climate change in some regions, are shown to increase pathogen exposure and contamination levels, jeopardizing human health and the resiliency of sanitation infrastructure [14].

The South Asia region has seen a continuous increase in the number of climate-related disasters, and the population is among the highest vulnerability for death or DALYs due to climatic hazards [1,15]. In 2023, the Intergovernmental Panel on Climate Change (IPCC) observed both an increase in extreme heatwaves and levels of heavy precipitation since the 1950s in the South Asia region [16]. These patterns are expected to persist with additional climatic hazards, resulting in adverse impacts on health and wellbeing, infrastructure and development, and the integrity and availability of the resources needed to meet basic human needs [16]. This documented increase in climate-related disasters is happening parallel to rapid population growth in the region, which is projected to increase by 800 million people by 2050 [17].

Bangladesh, a coastal country in South Asia bordering the Indian Ocean, is experiencing an acute combination of climate change impacts and public health risks. The Asian Climate Security Model determined that Bangladesh was the most vulnerable country in the region in terms of climate security and ranks second in terms of population at risk for mass death [15]. Additionally, Bangladesh lacks stable water, sanitation, and hygiene (WASH) infrastructure and practices that limit the transmission of infectious diseases. This has led to severe burdens of malnutrition for young children, resulting in decreased immunity and a greater susceptibility to illness [18]. The burden of disease in this region is disproportionate to its health care infrastructure, which lacks the adequate capacity and technology to successfully treat and cure the population, specifically children [19].

Aa a result of this confluence of climate variability, weakened health systems, and poor WASH infrastructure, a substantial body of literature exists that reveals complex interactions between these factors and their collective impacts on child diarrheal disease in Bangladesh. Previous studies have demonstrated the significant impacts of climate change on increasing waterborne diseases in Bangladesh, including diarrheal disease, resulting from rainfall extremes, temperature fluctuations, and flooding [7,20,21]. Additional research on environmental factors in Bangladesh, such as land cover, have demonstrated an association between diarrheal disease and land use change, urbanization, and decreased tree canopy through altered water management practices, decreased freshwater discharge, and access to clean water due to environmental degradation [22,23,24]. Beyond environmental influences on diarrheal disease in Bangladesh, the strong body of literature demonstrates an association with weak health infrastructure and access to improved WASH systems. Specifically, tubewell construction in Bangladesh has been successful in reducing the burden of diarrheal disease, but the quality and depth of these tubewells varies along with their effectiveness [25,26]. Similarly, access to health care, education, and safe sanitation have all been documented to be associated with diarrheal disease in this context [27,28,29]. Yet, despite the wealth of evidence supporting an association between environmental, structural, and social determinants of diarrheal disease in Bangladesh, these risks do not occur in isolation.

However, limited evidence exists on the nexus dynamics between these co-occurring risk factors beyond traditional statistical techniques that consider secondary predictors of diarrheal disease. Effective and efficient public health measures require population-level, landscape scale approaches to monitor and curb disease spread in Bangladesh and other LMICs. Traditional methods of collecting environmental and health data result in high logistical and cost constraints which lead to limitations in data collection in specific geographic areas and smaller sample sizes [30]. To our knowledge, only one other recent study has applied machine learning techniques to disentangle the many predictors of diarrheal disease in Bangladesh [31]. This study succeeds in highlighting the utility of machine learning in providing a data-driven foundation for policymakers to design targeted public health interventions in areas of overlapping risk, such as Bangladesh. Our study builds upon and goes beyond this foundation to include spatial environmental data, such as precipitation and temperature, on a longitudinal scale. These remote sensing approaches to acquiring and combining environmental and health outcomes data highlight an opportunity to leverage geospatial and publicly available datasets to improve analysis and outcomes. While standard epidemiological approaches remain useful, these large spatial datasets require new computational techniques that can identify relationships embedded in large complex datasets. Machine learning models, such as Random Forest classifiers, are well suited to this challenge.

We therefore address the following research question using machine learning techniques: what social, economic, and environmental factors influence DD and do they differ at the household and village level across Bangladesh?

## 2. Methods

### 2.1. Data Preparation

For more than 30 years, the Demographic and Health Surveys (DHS) Program of the United States Agency for International Development has conducted hundreds of surveys in more than 90 countries aimed at collecting critical representative data on the health and nutrition of surveyed populations [31]. The DHS accumulate data at the household level, where respondents are adult members belonging to the household (HH). Each HH is assigned two IDs by the DHS: one is unique to the HH and the other defines the HH group. These DHS-assigned groups are proximity based and have been likened to villages in other studies [32], which is how they will be referred to hereafter. The organization of the survey data at these two identifying levels allows for analyses to be conducted at both the household and village scales.

**Figure 1:**
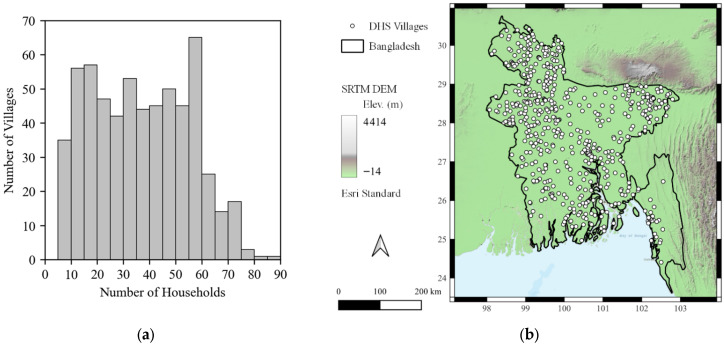
(**a**) Distribution of number of households per village is nearly uniformly distributed. (**b**) Bangladesh with the 600 DHS villages shown.

The DHS survey data used in this study consist of 21,779 survey responses from 600 DHS-defined groups, or villages, collected in the years 2004, 2007, and 2011 across the country of Bangladesh. The distribution in the number of households in each village is shown in Figure 1a which ranged from 5 to 86, with an average of about 36. The distribution appears to be bimodal, with one peak around the range of 10-25 households/village and another around the range of 55–60 households/village. The distribution skews to the right with a few villages having greater than 70 households/village.

The approximate locations of the villages are shown in Figure 1b. The DHS Program randomly offsets the locations of the survey villages to preserve confidentiality of the respondents, known as geo-masking. Urban locations are offset by 0–2 km and rural locations by 0–5 km, with an additional 1 percent of rural households further offset by 0–10 km [33]. The points shown in Figure 1b have a radius of 10 km to demonstrate the scale of the maximum potential offset relative to the size of Bangladesh. The DHS Program has conducted simulation studies aimed at investigating the impact of the random offset on spatial or distance-based analyses and found the impact of such geo-masking procedures to be negligible [34]. However, the authors of this study acknowledge that it may introduce some amount of uncertainty into the analysis. In addition to DHS Program responses, geographic (e.g., forest cover loss) and antecedent weather variables, or factors (e.g., slope, temperature, precipitation, and population density) were computed using the average within the 10 km buffer around each published village location [12]. Forest cover was calculated from the Hansen Global Forest Change dataset [35], while the global Climate Hazards Group InfraRed Precipitation and Temperatures with Stations datasets (CHIRPS and CHIRTS, respectively) [36], were used to calculate average monthly precipitation and temperature within a 10 km buffer around the published village location [12]. The survey dates were also classified into the four ordinal climatic seasons for Bangladesh: dry winter (December–February), pre-monsoon (March–May), monsoon (June–September), and post-monsoon (October–November) [32].

Features underwent an initial screening for model inclusion. Using knowledge from previous research regarding common indicators of DD [4,6,7,8,9,10,11,12,13,18,37], 30 features were selected from the complete list of survey features; the factors that were excluded were largely dietary in nature (N = 191). The selected features whose observations were greater than 15% missing were further removed from the analysis. Missing values in the remaining features were then imputed using Random Forest imputation from the ‘missForest’ package in R [38]. The algorithm utilizes non-parametric methods well-suited for mixed data types to impute missing values by way of Random Forest prediction [38].

Some data features are only present at the HH level (i.e., child’s age, mother’s education level, and diarrheal disease prevalence) while others differ only at the village level (i.e., weather-related features, population density, and average elevation). For village-scale features, all HHs belonging to the same village share the same value for these features. For village-level analyses, features that are variable at the HH level are aggregated to the village level using one of three methods depending on the data type. For features that are binary variables at the HH level, the prevalence of the feature is computed at the village level. For features that are continuous variables at the HH level, an average value is computed at the village level. For features that are categorical at the HH level, we selected the most frequent category to represent the feature at the village level. The final 22 features used in this study are shown in Table 1 including summary statistics and the aggregation method used at the village level if applicable.

### 2.2. Random Forest Classifier Models

**Figure 2:**
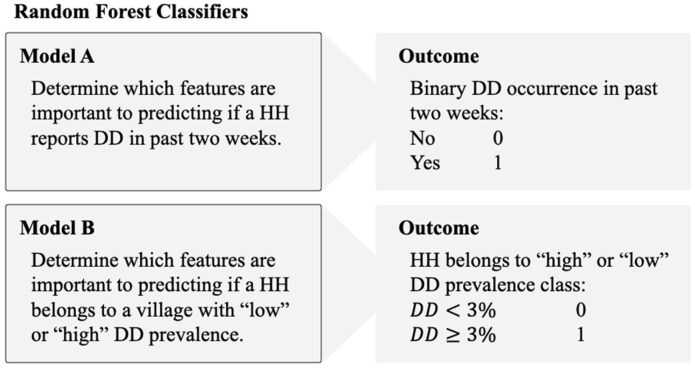
Depiction of the two models used in this study and their respective datasets and objectives.

Using the Random Forest (RF) classifier, two models were created for this study. The first, termed Model A in associated figures, used all HH survey responses (N = 21,779) with the response variable being the binary coding of whether a child in the HH has experienced DD in the previous two weeks. The second, termed Model B, also used all HH-level survey responses with each HH assigned to either the “high” DD prevalence or the “low” DD prevalence group. Descriptions of the datasets, models, and model objectives are shown in Figure 2.

**Figure 3:**
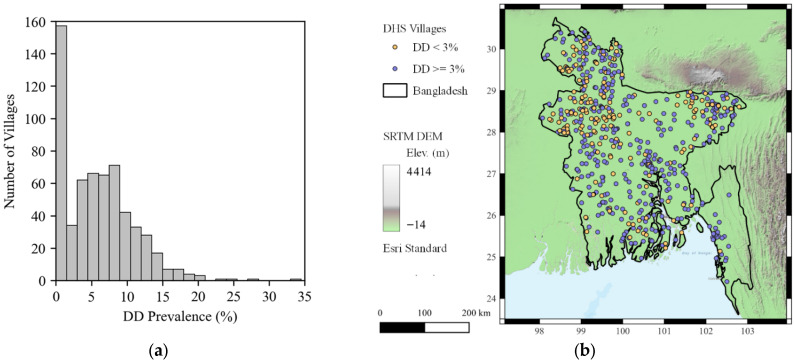
(**a**) Distribution of DD prevalence in villages, with 156 villages reporting a prevalence of zero. (**b**) DHS village locations coded by DD threshold.

To determine the three percent threshold for sub-setting the data into “high” or “low” groups, DD prevalence was computed for each of the 600 villages in Bangladesh as the ratio of households that report DD to the total number of households in that village. This ratio is expressed as a percentage in the histogram shown in Figure 3a depicting the distribution of DD prevalence in the villages.

There were N = 156 DHS villages with no households reporting DD, thus a prevalence of 0.0 percent. The highest DD prevalence for any village was 33 percent of households, while the mean prevalence across all villages was 5.9 percent. A threshold of 3.0 percent was used to divide the villages into the “low” (<3.0 percent) and “high” (≥3.0 percent) groups, resulting in an approximately 20–80 percent split of the data into the “low” (N = 179 villages) and “high” (N = 421 villages) DD groups, respectively. The 3.0 percent threshold was selected based on an inspection of the distribution of DD prevalence, shown in Figure 3a. The distribution is bimodal, with one peak below 3.0 percent and another around the range of 4.0–8.0 percent. The 3.0 percent cutoff represents the trough between these two modes. The locations of villages classified using the 3.0% threshold are shown in Figure 3b. Villages with “low” prevalence tend to be concentrated in the Northeastern and Northwestern portions of the country, while villages with “high” prevalence appear to be distributed across the country, with some concentrated areas in the Southeastern coast and population centers around the lower Padma River. Sample size information for each of the subsets is shown in Table 2. Of the 179 villages in the “low” DD group, 156 reported DD prevalence of 0.0 percent. Like the distribution of the number of HHs in villages shown in Figure 1a, the distribution of HHs in villages in the subsets are also nearly uniform and there did not appear to be a relationship between the number of households in a village and DD prevalence.

To train the two Random Forest models, the data were randomly divided into training and testing subsets using a 70/30 training/testing split. The trained models were used to make predictions on the test data, and the performance was evaluated using accuracy rates. Based on the test error performance metric, the optimal number of trees was found to be 300 for both Models A and B. To prevent overfitting and make the model more generalizable, the minimum number of samples required to perform a split in the tree was increased until the misclassification error on both the testing and training set was similar. Factors are presented in order of importance with associated SHAP values to aid interpretability [39,40].

## 3. Results

### 3.1. Model Performance

**Figure 4:**
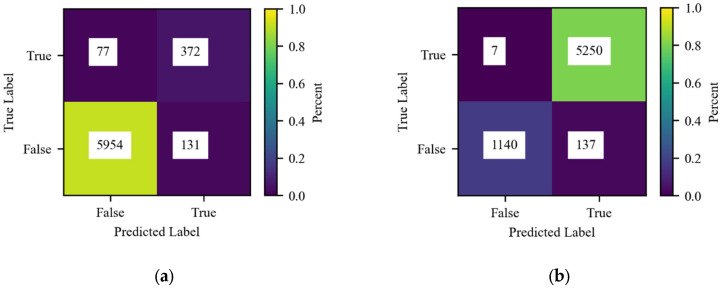
Confusion matrices for (**a**) Model A and (**b**) Model B where “True” labels indicate positive DD at the household (Model A) or village (Model B) level.

Both models A and B performed well, with accuracies of approximately 92% and 94%, respectively. Additional classification metrics (true and false positive rate and precision) are shown in Table 3. Confusion matrices for both models are shown in Figure 4. In both cases, the majority of misclassified cases were predicted to be positive for DD including 63% of misclassifications at the HH level and 95% at the village level. While false negatives may result in conclusions or policy recommendations that miss key relationships, false positives are more likely to result in more conservative conclusions and protective policy recommendations.

### 3.2. Interpretation of SHAP Values

**Figure 5:**
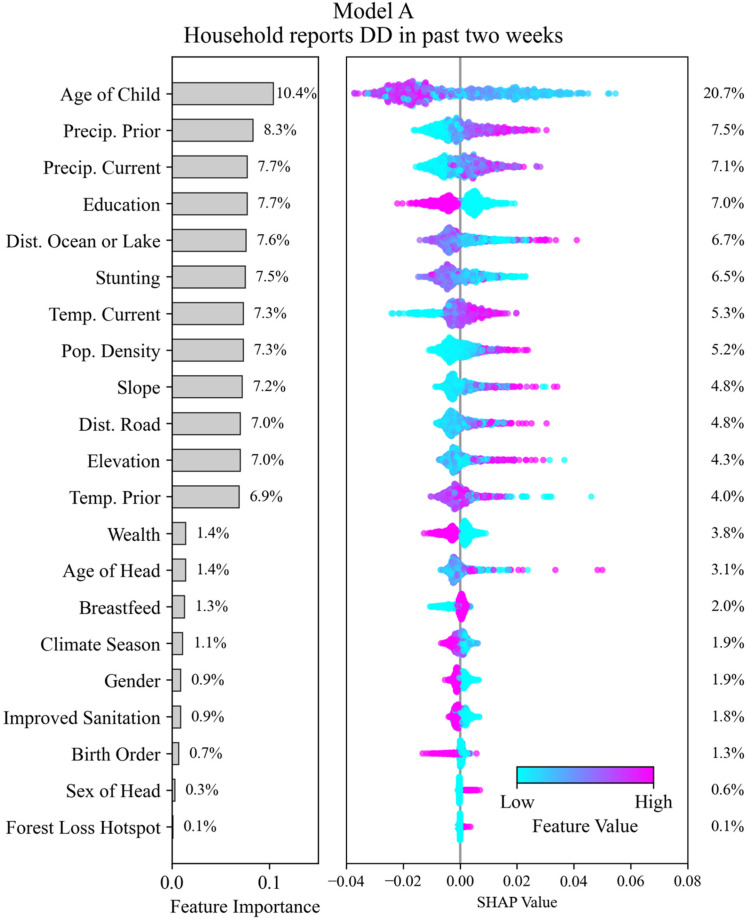
(**left**) Feature importance for Model A associated with the occurrence of a household reporting DD in the past two weeks determined using Gini Impurity. (**right**) SHAP values for corresponding features. The feature contribution percentage, computed based on absolute SHAP value, is shown on the right.

**Figure 6:**
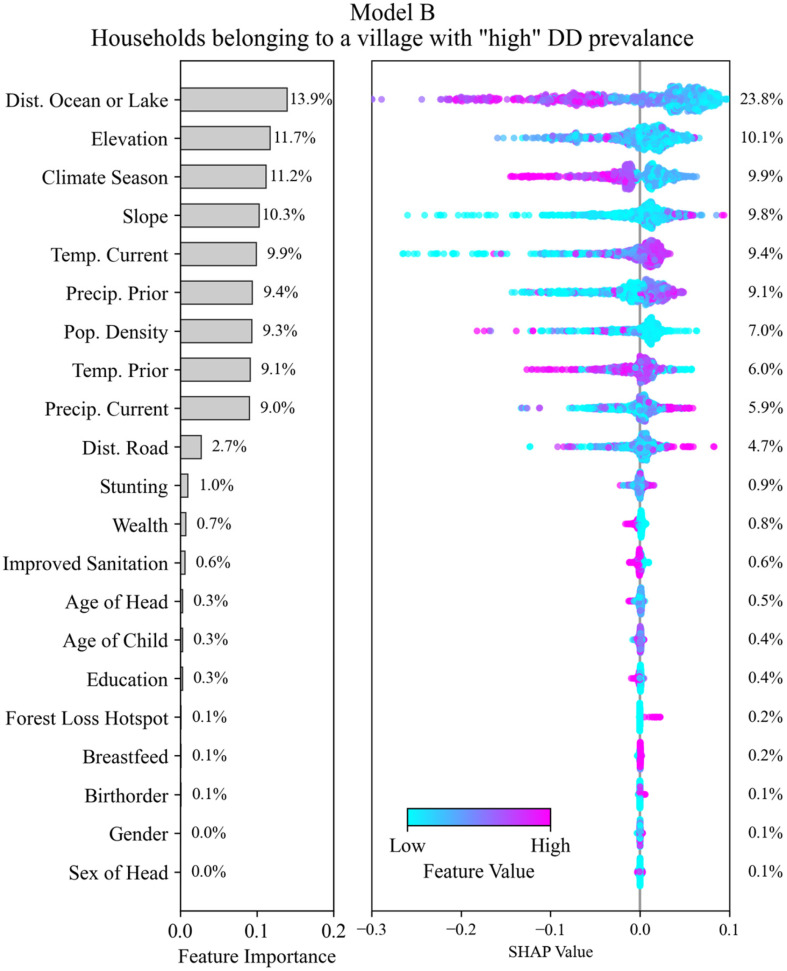
(**left**) Feature importance from Model B determining if a household belongs to a village with “high” DD prevalence determined using Gini Impurity. (**right**) SHAP values for the corresponding features. The feature contribution percentage, computed based on absolute SHAP value, is shown on the right.

Interpretation of the SHAP values helps to determine which features in the dataset (and their relative value ranges) are associated with the model outcomes. For each model, we plotted the SHAP values for each feature along the *x*-axis of the beeswarm plot (Figure 5 and Figure 6). The beeswarm plot represents the SHAP values associated with one outcome class: in Figure 5 the outcome class is “DD reported in past two weeks” and for Figure 6 the outcome class is “HH belongs to village with high DD prevalence”. The vertical spread of the points helps to show the density of points with similar SHAP values. The color indicates the relative value. A SHAP value of zero indicates the base value for the observations (feature values associated with a HH) that correspond to the outcome class. Positive SHAP values indicate that a feature (over a specific range of values) favors classifying an HH into the outcome class, and negative values indicate that it favors not being classified into the outcome class. The magnitude of the SHAP value indicates the relative strength of the contribution, and features are ordered on the *y*-axis by their mean contribution. After the model has been trained, we conducted SHAP analysis on a testing data subset of HHs (N = 5000).

### 3.3. Model A—Prediction of DD Outcomes at the Household Level

Feature selection was performed on all 23 features in Table 1 to identify the strongest predictors of DD outcomes at the household level based on whether a child in the HH had experienced DD in the two weeks prior to the DHS survey. Figure 5 shows the feature importance ranking and the associated SHAP values [39,40]; a higher importance value is indicative of stronger predictive power. Feature importance was determined using Gini Impurity, and the importance value shown for a feature represents its contribution to the model’s output. Note that the SHAP values are specific to a target outcome class: in the case of Figure 5 the outcome class is that a household reported DD within the two weeks prior to the survey. The contribution percentage of each feature determined using the SHAP analysis is also shown to the right. Note that the contribution percentage determined using both Gini Impurity and SHAP analysis can be different because they are two distinct methods of estimating feature attribution.

Age of child is the most important feature, with the next 11 features having similar importance and comprising geographic, physical, and climatological features. We observe a significant decrease in feature importance after the 12th feature (temp. prior). Notable climatological features include prior (ranked 2nd) and current month temperature (ranked 3rd) and precipitation (ranked 7th). Notable geographic features include slope (ranked 9th), elevation (ranked 11th), and distance to large water bodies (ranked 5th) and roadways (ranked 10th). Population density (ranked 8th) was the only socio-demographic factor that emerged as significant. Additional factors including Wealth, Gender, Improved Sanitation, Climate Season, and Forest Loss Hotspot were not deemed significant.

The beeswarm plots help with interpreting each feature’s influence on the model. The most important feature, age of child, for example, has positive SHAP values associated with “low” to “medium-low” feature values (i.e., occurrence of a household reporting DD in the past two weeks) while higher feature values tend to have negative SHAP values. This suggests that younger children are more likely to have experienced DD in the two weeks prior to the survey. For binary features, such as education, the effect can be clearer: we see that “low” values of the feature (a “0” indicating that the respondent does not have higher or secondary education) have positive SHAP values, suggesting that the lack of higher or secondary education increases the likelihood of the household reporting DD in the past two weeks. This method of interpretation showcases which features, and the general ranges and magnitude of impact of those features, are associated with an increased likelihood of reporting DD. These data show that medium to high values of prior and current precipitation lead to higher likelihood of DD in the HH, along with higher values of population density, slope, distance to road, and elevation. Meanwhile, low or high distance to lakes may lead to higher DD incidence and low temperatures may lower DD risk at the household level.

Most of the top 12 important features have nearly the same importance, except for age of child. This suggests that the prediction of DD occurrence at the household level may involve complex non-linear interactions between these features. The difference in the impact of feature value on the model output of some high importance features, such as childhood stunting (a height for age <−2 z-scores below the median of the WHO Child Growth Standards) [41,42], is not immediately clear from this visualization alone. This is possible because this evaluation method does not consider potential interactions between features, and stunting may interact strongly with other features. Many low-importance features show well-defined splits between high and low feature values (i.e., improved sanitation, wealth, and birth order), however the impact on the model output from these features is small relative to more important features.

### 3.4. Model B—Prediction of Households Belonging to High or Low DD Occurrence Villages

All features in Table 1 were tested to determine the strongest predictors of HH membership in a “high” or “low” DD occurrence village. Figure 6 shows the importance ranking of the model and associated SHAP values. Note that the SHAP values are specific to one outcome class: in the case of Figure 6, the outcome class is that a household belongs to a village that has high DD prevalence (more than 3% of HHs within the village reporting DD within the two weeks prior to the survey).

Similarly to Model A, there is a clear cutoff in feature importance, which in this case is after the ninth feature (precip. current). The most important features are geographic or climate related with no household level socio-demographic features (i.e., wealth and education) being significant in predicting household membership to a village of high DD prevalence. Of the geographic features, distance to the ocean or large water bodies (ranked 1st) and elevation (ranked 2nd) are the two most important features along with slope (ranked 4th). SHAP values indicate that large distance values from the ocean or large water bodies decrease the likelihood of an HH belonging to a high DD prevalence village while the effect of elevation is less clear. Lower slope values, which may be an indicator of flood risk, decrease the likelihood of an HH belonging to a high DD village.

Climatological factors were secondarily important with climate season (ranked 3rd) the most important and appearing to have a bifurcation effect with pre-monsoon seasons (low values), increasing the likelihood of an HH belonging to a high DD prevalence village and monsoon and post-monsoon seasons (high values) decreasing that likelihood. Current temperature (ranked 5th) and antecedent temperature (ranked 8th) were also significant with higher current temperatures and lower prior temperatures increasing the likelihood of an HH belonging to a high DD village based on SHAP value distributions. Past and current precipitation (ranked 5th and 9th, respectively), meanwhile suggest that higher values of each increase the likelihood of an HH being in a high DD prevalence village. Population density (ranked 7th) constituted the final significant factor with lower values increasing the likelihood of an HH belonging to a high DD prevalence village.

## 4. Discussion

These findings highlight the complex interplay of individual, environmental, and socio-demographic factors in the prevalence of diarrhea at multiple scales of analysis. Understanding the difference and controllability of these factors across scales can better inform potential public health intervention strategies. The evidence supports the research question by showing that differences exist in the factors driving DD prevalence at the HH and village scales. The most important distinction between the HH and village level findings is the importance of socio-demographic characteristics at the HH level, while climatological and geographic features alone emerged as significant predictors at the village level. At the HH level, the age of child was the most important factor, with younger children most vulnerable to experiencing DD in the past two weeks, consistent with the previous literature [1,4]. This finding suggests that prenatal and pediatric interventions associated with an improved health care system infrastructure and quality may be most effective in part by increasing risk awareness among household adults [18,19]. Features such as education and stunting, meanwhile, likely require similarly broad systematic interventions to promote general education and improved access to balanced diets.

At the village level, the findings suggest some support for the concentration–dilution hypothesis with villages proximate to large waterbodies most at risk, particularly during the dry winter and pre-monsoon seasons when DD pathogens may concentrate in available water supplies [9,11]. Furthermore, lower slopes were found to decrease the likelihood of a household belonging to a high DD prevalence village, suggesting that some amount of flushing under flood conditions consistent with past research [14], though this was not a uniform finding across the dataset based on the SHAP value distribution. Interestingly, at the village scale, households in low population density villages were more at risk, while at the household level, that relationship was flipped. This was the only significant factor that clearly changed signs between the two scales of analysis, though is consistent with past studies which have found a complex interplay of factors across the urban–rural continuum [10,13].

On longer time scales, the findings further suggest that changing climate conditions, including increased extreme precipitation patterns and warming temperatures, have the potential to impact DD prevalence. At the village scale, these relationships are complex and secondary to geographic factors; however, at the HH level, both prior and current month precipitation are second only to age of child in importance to predicting DD in the last two weeks. These appear to have a more strongly dichotomous SHAP value relationship with higher values increasing DD prevalence.

Notably, missing from the determined significant features were land use change, improved sanitation, and economic status. Land use change, as captured by the forest loss hotspot factor, was not found to be significant at either the HH or village level. However, Bangladesh may represent a unique case [15], having relatively low levels of forest coverage over time and therefore low levels of forest cover change. The hotspots, therefore, are likely to represent outliers of land cover change that had a relatively small influence on the models, which are less sensitive to outliers than other statistical approaches. Decision tree algorithms, such as Random Forest, bypass some of the assumptions and restrictions of, for example, logistic regression, a common methodological approach in public health and epidemiological analyses. Forest coverage loss may, however, be a significant driver of DD in neighboring countries, which have historically maintained higher levels of forest land cover and may be seeing significant rates of loss in recent years and decades. Similarly, diarrheal disease has many risk factors and etiologies that were not able to be considered in this analysis, which may result in an overestimation of feature importance in our models. However, we were successfully able to include many of the most prominent drivers of diarrheal disease identified in the literature, thereby minimizing this limitation. Regional-level analysis or paired-country analysis is required to tease apart these larger-scale signals and associated policy implications to improve forecasts and intervention design. Improved sanitation and economic status were also not found to have high importance. While at both the HH and village scale lower values of each were associated with increased risk of DD, the importance of these factors was much smaller than other features.

## 5. Conclusions

These findings suggest that a multi-tiered forecasting and prediction approach may be most appropriate for targeting resources. While the village-level findings may be most useful in developing forecasts that predict disease outbreak, the findings at the HH level may be better suited to targeting resources to improve outcomes across timescales. Furthermore, on-the-ground research is needed to assess the functional mechanisms of significant factors.

## Figures and Tables

**Table 1:** Summary statistics for all features used in the study, including description, units, scale of calculation, and the aggregation method for bringing household-level features to the village level.

Feature	Description	Type	Scale	Mean (S.D.) ^a^	Aggregation Method ^b^
Socio-Demographic Features					
Age of Child	Age of child in months.	Continuous	HH	29.9 (17.2)	Average
Stunting	Height/age standard deviations according to the WHO.	Continuous	HH	−75.5 (213.3)	Average
Age of Head	Age of the head of household in years.	Continuous	HH	41.7 (14.1)	Average
Pop. Density	Average population density in 5 km radius.	Continuous	Village	2814 (6320)	n/a
Education	Respondent has higher or secondary education.	Binary	HH	42%	Prevalence
Gender	Gender of child (male).	Binary	HH	49%	Prevalence
Improved Sanitation	Improved sanitation based on WHO definitions.	Binary	HH	53%	Mode
Breastfeed	Respondent is currently breastfeeding.	Binary	HH	68%	Prevalence
Wealth	Respondent is in upper two quintiles of wealth.	Binary	HH	39%	Prevalence
Birth Order	Birth order of child experiencing diarrheal disease.	Categorical (1–5)	HH	1.2 (0.45)	Mode
Sex of Head	Sex of the head of household (male).	Binary	HH	8%	Prevalence
Climate and Geographic Features					
Precip. Previous	Total precipitation (mm) in month prior to survey.	Continuous	Village	265.9 (254.9)	
Precip. Current	Total precipitation (mm) of month of survey.	Continuous	Village	286.1 (271.5)	n/a
Temp. Previous	Average temperature (°C) of month prior to survey.	Continuous	Village	30.98 (2.8)	n/a
Temp. Current	Average temperature (°C) of month of survey.	Continuous	Village	30.96 (2.7)	n/a
Climate Season	Climate season at time of survey. (1 = dry winter; 2 = pre-monsoon; 3 = monsoon; 4 = post-monsoon).	Categorical (1–4)	Village	2.4 (0.96)	n/a
Dist. Ocean or Lake	Distance (m) to ocean or lake.	Continuous	Village	248,781 (171,040)	n/a
Elevation	Average elevation (m) of the village.	Continuous	Village	14.22 (11.4)	n/a
Slope	Average slope within 5 km radius of village.	Continuous	Village	0.12 (0.18)	n/a
Dist. Road	Distance (m) to nearest road.	Continuous	Village	2214 (2341)	n/a
Forest Loss Hotspot	Village located in a forest loss hotspot.	Binary	Village	2%	n/a
Health Outcome Features					
DD HH	Child in household has experience diarrheal disease in the past two weeks.	Binary	HH	7%	Prevalence
DD Village	Diarrheal disease experienced in ≥3% village HHs.	Binary	Village	80%	

Notes: ^a^ Mean and standard deviation (S.D.) values calculated across all households. ^b^ Aggregation method for bringing household-level features to the village level.

**Table 2:** Summary Statistics for Select factors for.

Dataset	Sample Size
Household-Level	21,779 households
Village-Level	600 villages
“Low” DD group	4257 households in 179 villages
“High” DD group	17,522 households in 421 villages

**Table 3:** Classification metrics for both models.

Metric	A	B
Recall (true positive rate)	82.9%	99.9%
False positive rate	2.2%	10.7%
Precision	74.0%	97.5%

## Data Availability

DHS data are available at: https://dhsprogram.com/data/ (accessed on 13 October 2024). The datasets used and analyzed during the current study are available from the corresponding author on reasonable request.

## Abbreviations

DD	Diarrheal Disease.
DALYs	Disability-Adjusted Life Years
LMICs	Low- and Middle-Income Countries
WHO	World Health Organization
IPCC	Intergovernmental Panel on Climate Change
WASH	Water, Sanitation, and Hygiene
DHS	Demographic and Health Surveys
HH	Household
IDs	Identifiers
CHIRPS	Climate Hazards Group InfraRed Precipitation with Stations
CHIRTS	Climate Hazards Group InfraRed Temperature with Stations
RF	Random Forest
SHAP	Shapley Additive Explanations

## References

[1] Vos T., Lim S.S., Abbafati C., Abbas K., Abbasi M., Abbasifard M., Abbasi-Kangevari M., Abbastabar H., Abd-Allah F., Abdelalim A. (2020). Global burden of 369 diseases and injuries in 204 countries and territories, 1990–2019: A systematic analysis for the Global Burden of Disease Study 2019. Lancet.

[2] Bakir H., Hadi M., Jurdi M. (2017). Towards a renewed public health regulatory and surveillance role in water, sanitation and hygiene. East. Mediterr. Health J..

[3] Hodge J., Chang H.H., Boisson S., Collin S.M., Peletz R., Clasen T. (2016). Assessing the Association between Thermotolerant Coliforms in Drinking Water and Diarrhea: An Analysis of Individual-Level Data from Multiple Studies. Environ. Health Perspect..

[4] Demissie G.D., Yeshaw Y., Aleminew W., Akalu Y. (2021). Diarrhea and associated factors among under five children in sub-Saharan Africa: Evidence from demographic and health surveys of 34 sub-Saharan countries. PLoS ONE.

[5] Meki C.D., Ncube E.J., Voyi K. (2022). Frameworks for mitigating the risk of waterborne diarrheal diseases: A scoping review. PLoS ONE.

[6] Mertens A., Balakrishnan K., Ramaswamy P., Rajkumar P., Ramaprabha P., Durairaj N., Hubbard A.E., Khush R., Colford J.M., Arnold B.F. (2019). Associations between High Temperature, Heavy Rainfall, and Diarrhea among Young Children in Rural Tamil Nadu, India: A Prospective Cohort Study. Environ. Health Perspect..

[7] Levy K., Woster A.P., Goldstein R.S., Carlton E.J. (2016). Untangling the Impacts of Climate Change on Waterborne Diseases: A Systematic Review of Relationships between Diarrheal Diseases and Temperature, Rainfall, Flooding, and Drought. Environ. Sci. Technol..

[8] Carlton E.J., Eisenberg J.N.S., Goldstick J., Cevallos W., Trostle J., Levy K. (2014). Heavy Rainfall Events and Diarrhea Incidence: The Role of Social and Environmental Factors. Am. J. Epidemiol..

[9] Levy K., Hubbard A.E., Nelson K.L., Eisenberg J.N.S. (2009). Drivers of Water Quality Variability in Northern Coastal Ecuador. Environ. Sci. Technol..

[10] Kulinkina A.V., Mohan V.R., Francis M.R., Kattula D., Sarkar R., Plummer J.D., Ward H., Kang G., Balraj V. (2016). Seasonality of water quality and diarrheal disease counts in urban and rural settings in south India. Sci. Rep..

[11] Kraay A.N.M., Man O., Levy M.C., Levy K., Ionides E., Eisenberg J.N.S. (2020). Understanding the Impact of Rainfall on Diarrhea: Testing the Concentration-Dilution Hypothesis Using a Systematic Review and Meta-Analysis. Environ. Health Perspect..

[12] Herrera D., Ellis A., Fisher B., Golden C.D., Johnson K., Mulligan M., Pfaff A., Treuer T. (2017). Upstream watershed condition predicts rural children’s health across 35 developing countries. Nat. Commun..

[13] Kattula D., Francis M.R., Kulinkina A.V., Sarkar R., Mohan V.R., Babji S., Ward H.D., Kang G., Balraj V., Naumova E.N. (2015). Environmental predictors of diarrhoeal infection for rural and urban communities in south India in children and adults. Epidmeiol. Infect..

[14] Gong L., Hou S., Su B., Miao K., Zhang N., Liao W., Zhong S., Wang Z., Yang L., Huang C. (2019). Short-term effects of moderate and severe floods on infectious diarrheal diseases in Anhui Province, China. Sci. Total Environ..

[15] Busby J., Smith T.G., Krishnan N., Wight C., Vallejo-Gutierrez S. (2018). In harm’s way: Climate security vulnerability in Asia. World Dev..

[16] IPCC (2023). Climate Change 2023: Synthesis Report.

[17] United Nations (2024). World Population Prospects 2024: Summary of Results.

[18] Muhammad F., Chowdhury M., Arifuzzaman M., Chowdhury A.A. (2016). Public Health Problems in Bangladesh: Issues and challenges. South East Asia J. Public Health.

[19] Sultana M., Mahumud R.A., Sarker A.R. (2015). Emerging Patterns of Mortality and Morbidity in District Level Hospitals in Bangladesh. Ann. Public Heal Res..

[20] Harris A.M., Chowdhury F., Begum Y.A., Khan A.I., Faruque A.S.G., Svennerholm A.-M., Harris J.B., Ryan E.T., Cravioto A., Calderwood S.B. (2008). Shifting Prevalence of Major Diarrheal Pathogens in Patients Seeking Hospital Care during Floods in 1998, 2004, and 2007 in Dhaka, Bangladesh. Am. J. Trop. Med. Hyg..

[21] Schwartz B.S., Harris J.B., Khan A.I., Larocque R.C., Sack D.A., Malek M.A., Faruque A.S.G., Qadri F., Calderwood S.B., Luby S.P. (2006). Diarrheal epidemics in Dhaka, Bangladesh, during three consecutive floods: 1988, 1998, and 2004. Am. J. Trop. Med. Hyg..

[22] Kanan A.H., Pirotti F. (2022). A Comparative Assessment of Land Use-Land Cover Dynamics Between Bangladesh and Indian Sundarbans From 1975–2020: A Geospatial and Statistical-Based Approach. Int. Arch. Photogramm. Remote Sens. Spat. Inf. Sci..

[23] Moniruzzam M., Roy A., Bhatt C.M., Gupta A., An N.T.T., Hassan M. (2018). Impact Analysis of Urbanization on Land Use Land Cover Change for Khulna City, Bangladesh Using Temporal Landsat Imagery. Int. Arch. Photogramm. Remote Sens. Spat. Inf. Sci..

[24] Uddin I.M., Endres K., Parvin T., Bhuyian M.S.I., Zohura F., Masud J., Monira S., Hasan M.T., Biswas S.K., Sultana M. (2023). Food Hygiene and Fecal Contamination on the Household Compound are Associated with Increased Pediatric Diarrhea in Urban Bangladesh (CHoBI7 Program). Am. J. Trop. Med. Hyg..

[25] Wu J., van Geen A., Ahmed K.M., Alam Y.A.J., Culligan P.J., Escamilla V., Feighery J., Ferguson A.S., Knappett P., Mailloux B.J. (2011). Increase in diarrheal disease associated with arsenic mitigation in Bangladesh. PLoS ONE.

[26] Goel V., Bell G.J., Sridhar S., Islam M.S., Yunus M., Ali M.T., Khan M.A., Alam M.N., Faruque A., Kabir M.M. (2020). Considering Alternate Pathways of Drinking-Water Contamination: Evidence of Risk Substitution from Arsenic Mitigation Programs in Rural Bangladesh. Int. J. Environ. Res. Public Health.

[27] Colombara D., Khalil I., Rao P., Troeger C., Forouzanfar M.H., Riddle M.S., Mokdad A.H. (2016). Chronic Health Consequences of Acute Enteric Infections in the Developing World. Am. J. Gastroenterol. Suppl..

[28] Akter J., Islam M.R., Akter S., Rahman M.M., Hossain F., Anam M.R., Alam M.A., Sultana P., Rashid S. (2022). Equity in access to safely managed sanitation and prevalence of diarrheal diseases in Bangladesh: A national and sub-national analysis. BMC Infect. Dis..

[29] Kamal M., Tewabe T., Tsheten T. (2022). Individual and community-level factors associated with under-five diarrhea in Bangladesh: Evidence from Demographic and Health Survey 2014. Curr. Ther. Res. Clin. Exp..

[30] Mall R.K., Srivastava R.K., Banerjee T., Mishra O.P., Bhatt D., Sonkar G. (2019). Disaster Risk Reduction Including Climate Change Adaptation Over South Asia: Challenges and Ways Forward. Int. J. Disaster Risk Sci..

[31] DHS The DHS Program—Bangladesh: Standard DHS, 2022 Dataset. https://dhsprogram.com/data/dataset/Bangladesh_Standard-DHS_2022.cfm?flag=0.

[32] Alamgir M., Shahid S., Hazarika M.K., Nashrrullah S., Harun S.B., Shamsudin S. (2015). Analysis of Meteorological Drought Pattern During Different Climatic and Cropping Seasons in Bangladesh. JAWRA J. Am. Water Resour. Assoc..

[33] Perez-Haydrich C., Warren J.L., Burgert C.R., Emch M.E. Guidelines on the Use of DHS GPS Data. https://dhsprogram.com/publications/publication-SAR8-Spatial-Analysis-Reports.cfm.

[34] Burgert C.R., Colston J., Roy T., Zachary B. Geographic Displacement Procedure and Georeferenced Data Release Policy for the Demographic and Health Surveys. https://dhsprogram.com/publications/publication-SAR7-Spatial-Analysis-Reports.cfm.

[35] Hansen M.C., Potapov P.V., Moore R., Hancher M., Turubanova S.A., Tyukavina A., Thau D., Stehman S.V., Goetz S.J., Loveland T.R. (2013). High-Resolution Global Maps of 21st-Century Forest Cover Change. Science.

[36] Funk C., Peterson P., Landsfeld M., Pedreros D., Verdin J., Shukla S., Husak G., Rowland J., Harrison L., Hoell A. (2015). The climate hazards infrared precipitation with stations—A new environmental record for monitoring extremes. Sci. Data.

[37] Bauch S.C., Birkenbach A.M., Pattanayak S.K., Sills E.O. (2015). Public health impacts of ecosystem change in the Brazilian Amazon. Proc. Natl. Acad. Sci. USA.

[38] Stekhoven D.J., Bühlmann P. (2012). MissForest—Non-parametric missing value imputation for mixed-type data. Bioinformatics.

[39] Louhichi M., Nesmaoui R., Mbarek M., Lazaar M. (2023). Shapley Values for Explaining the Black Box Nature of Machine Learning Model Clustering. Procedia Comput. Sci..

[40] Rodríguez-Pérez R., Bajorath J. (2020). Interpretation of machine learning models using shapley values: Application to compound potency and multi-target activity predictions. J. Comput. Aided Mol. Des..

[41] Gleason K.M., Valeri L., Shankar A.H., Hasan M.O.S.I., Quamruzzaman Q., Rodrigues E.G., Christiani D.C., Wright R.O., Bellinger D.C., Mazumdar M. (2016). Stunting is associated with blood lead concentration among Bangladeshi children aged 2–3 years. Environ. Health.

[42] WHO Multicentre Growth Reference Study Group (2006). WHO Child Growth Standards: Length/Height-for-Age, Weight-for-Age, Weight-for-Length, Weight-for-Height and Body Mass Index-for-Age: Methods and Development.

